# Impact of Four Protein Additives in Cryogels on Osteogenic Differentiation of Adipose-Derived Mesenchymal Stem Cells

**DOI:** 10.3390/bioengineering6030067

**Published:** 2019-08-07

**Authors:** Victor Häussling, Sebastian Deninger, Laura Vidoni, Helen Rinderknecht, Marc Ruoß, Christian Arnscheidt, Kiriaki Athanasopulu, Ralf Kemkemer, Andreas K. Nussler, Sabrina Ehnert

**Affiliations:** 1 Siegfried Weller Research Institute, BG Unfallklinik Tuebingen, Department of Trauma and Reconstructive Surgery, Eberhard Karls University Tübingen, 72074 Tübingen, Germany; 2 Department of Applied Chemistry Reutlingen University, 72762 Reutlingen, Germany

**Keywords:** bone tissue engineering, cryogel, adipose-derived mesenchymal stem/stromal cells (Ad-MSCs), 3D-culture, scaffold, platelet-rich plasma (PRP), RGD, collagen, immune-cell conditioned medium

## Abstract

Human adipose-derived mesenchymal stem/stromal cells (Ad-MSCs) have great potential for bone tissue engineering. Cryogels, mimicking the three-dimensional structure of spongy bone, represent ideal carriers for these cells. We developed poly(2-hydroxyethyl methacrylate) cryogels, containing hydroxyapatite to mimic inorganic bone matrix. Cryogels were additionally supplemented with different types of proteins, namely collagen (Coll), platelet-rich plasma (PRP), immune cells-conditioned medium (CM), and RGD peptides (RGD). The different protein components did not affect scaffolds’ porosity or water-uptake capacity, but altered pore size and stiffness. Stiffness was highest in scaffolds with PRP (82.3 kPa), followed by Coll (55.3 kPa), CM (45.6 kPa), and RGD (32.8 kPa). Scaffolds with PRP, CM, and Coll had the largest pore diameters (~60 µm). Ad-MSCs were osteogenically differentiated on these scaffolds for 14 days. Cell attachment and survival rates were comparable for all four scaffolds. Runx2 and osteocalcin levels only increased in Ad-MSCs on Coll, PRP and CM cryogels. Osterix levels increased slightly in Ad-MSCs differentiated on Coll and PRP cryogels. With differentiation alkaline phosphatase activity decreased under all four conditions. In summary, besides Coll cryogel our PRP cryogel constitutes as an especially suitable carrier for bone tissue engineering. This is of special interest, as this scaffold can be generated with patients’ PRP.

## 1. Introduction

The regeneration of bone to correct large bone defects after trauma or tumor surgery remains a significant issue in orthopedic and trauma surgery [1]. Treatment strategies require bone fixation in order to preserve skeletal architecture as well as filling the gap to allow bone bridging and finally healing [2]. Highly specialized surgical techniques, e.g., microvascular anastomosed autologous bone grafting, Masquelet technique, or Ilizarov external fixation with segmental bone transport, are established in order to repair large bone defects. However, these methods require large amounts of biologically active bony tissue, which while filling in the defect area provides mesenchymal stem/stromal cells (MSCs) essential for bone healing [3,4]. Thus, the lack of biologically active bone material is a critical issue [5]. Patients’ own bone for autologous transplantation is limited and allogenic transplantation of bone tissue from donors is always accompanied by immunological risks. Alloplastic synthetic bone filling materials can also be used to fill the defect area. However, often lacking osteo-inductive capacity, these filling materials carry the risk of failing. Mixing the material with platelet-rich plasma (PRP), bone marrow concentrate or reaming irrigation aspirate may increase healing success by recruiting resident MSCs and osteoblasts to the bone defect [6], a mechanism mediated by factors secreted from immune cells [7]. Tissue engineering tries to solve that issue by producing biologically active bone material for transplantation, which consists of the patients’ own stem cells on a scaffold that mimics the extracellular matrix of bone. With MSCs being the most transplanted type of cells, the number of clinical trials investigating the cell-therapeutic potential of MSCs continuously increases [8].

Facing strict regulations for transplantation, MSCs derived from bone marrow aspirates (B-MSCs) are predominantly used for bone regeneration. However, these cells also face limitations. Only a limited amount can be obtained in order to minimize the risk for side effects, i.e., anemia [9]. Thus, harvesting bone marrow is not only painful but may also be risky for the patient. Therefore, the use of adipose tissue as a possible resource for autologous MSCs should be considered [9,10]. Fat tissue contains large amounts of so-called adipose-derived MSCs (Ad-MSCs) [11]. In general, these cells can be obtained in larger amounts at a lower risk for the patient, due to the minimally invasive harvesting technique [10,12,13]. However, critical discussions on the osteogenic differentiation potential of Ad-MSCs persist. There are reports, showing that both Ad-MSCs and B-MSCs can be stimulated to form bone tissue when transplanted undifferentiated on bone scaffolds [14,15]. However, mechanical properties of newly formed bone tissue might favor the use of B-MSCs [16]. This may be for several reasons. Reports have shown that permanent psychological stress, increased donor age and extended cell cultures reduce the osteogenic differentiation potential of MSCs, via epigenetic mechanisms [17,18,19,20]. In addition, we could recently show, that donor site location (abdomen, hip, thigh, knee, and limb), has a critical impact on the osteogenic differentiation potential of Ad-MSCs [21]. Another critical factor affecting the osteogenic differentiation potential is the support material itself. In general, 3D cultures mimicking the three-dimensional environment of bone may favor the osteogenic differentiation of the applied MSCs [22]. Thus, 3D cultures seem to be more beneficial for the differentiation when compared to 2D cultures [23]. But 3D cultures also raise new challenges, i.e., proper adjustment of the physical characteristics of the 3D matrix to the human physiology, uniform cell seeding on the scaffold and adequate supply with nutrients [24], factors associated with each other. Regarding scaffold architecture, pore size, porosity, and stiffness are essential. Pore size and porosity not only regulate cell attachment and cell infiltration into the scaffold but also indirectly affect nutrient diffusion, a factor that may be actively influenced by applying medium flow to the cultures. Common scaffolds based on collagen are mostly flexible in order to pass on mechanical stimuli to the cells [25], a factor known to favor osteogenic differentiation. However, if the scaffold stiffness is too low, this may favor MSC differentiation toward adipocytes and not osteoblasts [26]. Thus, the choice of scaffold strongly affects the success of the culture. At the moment there are numerous different material sources and production processes being tested in relation to certain advantages and disadvantages [27,28]. Among the huge number of scaffolds available, cryogels stand out because of their porous structure, which effectively mimics the structure of spongy bone [29,30]. Various monomers can serve as a base for cryogels, which in combination with the freezing temperature affect the pore size and stiffness of the gels [29]. Besides the possible variability, cryogels are easy and cheap to produce and can be generated in different shapes. Poly(2-hydroxyethyl methacrylate) (pHEMA) is a common monomer for the generation of cryogels. It is often polymerized with methacrylate or bisacrylamide as cross-linkers. Resulting cryogels are frequently used as scaffolds for in vitro studies or carrier for tissue engineering approaches [31]. Regarding bone Kim et al. have shown that application of pHEMA based cryogels can improve healing of large bone calvarial defects in rabbits [32].

In this study we aimed at generating pHEMA based cryogels that successfully mimic both bone architecture (porosity, pore size and stiffness) and bone inorganic and organic matrix. Furthermore, we aimed at identifying the osteo-inductive potential of different protein additives for these scaffolds, e.g., collagen (Coll) which represents the most common scaffold material, RGD-peptides (RGD) which represents the best standardized scaffold coating, PRP which represents a clinically used additive for synthetic bone filling material, as well as immune-cell conditioned medium (CM) which provides chemokines and growth factors for osteoprogenitor cells.

## 2. Materials and Methods

Chemicals and reagents were obtained from Sigma Aldrich (Munich, Germany) or Carl Roth (Karlsruhe, Germany). Culture medium and its supplements were obtained from Sigma Aldrich.

### 2.1. Scaffold Manufacturing

Scaffolds were manufactured by polymerization of biocompatible monomers under frozen conditions, the so-called cryogel-technique. Briefly, pHEMA, bisacrylamide, water and the protein source were mixed and incubated for 30 min on ice. Afterwards di-sodium hydrogen phosphate buffer, glutaraldehyde, ammonium persulfate (APS), and tetramethylethylenediamine (TEMED) were added. Used volumes and concentrations are given in Table 1. The resulting reaction solution was thoroughly mixed and immediately distributed (2 mL per mold) into casting molds (inner diameter = 5 mm). Filled molds were instantly frozen for at least 17 h at −18 °C. To obtain scaffolds with a uniform size, the polymerized matrix was deep frozen for 1 h at −80 °C in order to ease slicing with a razor blade. The height of the resulting scaffold (3 mm) was determined by a spacer. To facilitate calcium phosphate (hydroxyapatite) precipitation sliced cryogels were immediately transferred to 1 M CaCl_2_ solution for 24 h (agitation). 

### 2.2. Physical Characterization of Scaffolds

#### 2.2.1. Pore Size

The organic matrix components of the scaffold were stained with sulforhodamine B (SRB) to visualize the scaffold structure. Briefly, scaffolds were stained for 30 min with 0.08% SRB in 1% acetic acid. Unbound SRB was washed off the scaffolds with 1% acetic acid [34]. The remaining bound SRB emits a red fluorescent signal, which was detected with a fluorescence microscope (Evos Fl, Thermo Fisher Scientific, Karlsruhe, Germany). Using the ImageJ software (NIH, Bethesda, MD, USA) pore size and shape were determined. Per scaffold, 3 images (each 10 representative pores) were measured, by two independent investigators in a blinded fashion.

#### 2.2.2. Scanning Electron Microscopy (SEM)

The surface morphology as well as the fracture surface of different freeze-dried scaffolds were observed using scanning electron microscopy (Zeiss DSM 962, Zeiss, Oberkochen, Germany). The samples were cut into pieces, mounted on aluminum stubs using a double-sided adhesive carbon tape and sputter-coated with gold prior to examination (*t* = 3 min and 16 mA).

#### 2.2.3. Porosity and Swelling Ratio

In 2005 Shimizu et al. developed a formula to determine the porosity of scaffolds based on scaffolds’ wet and dry weight [35]. By slightly modifying this formula the swelling ration can be determined. As described before, these formulas were used to characterize the scaffolds [36]. Using an analytical balance [g] the dry and wet (scaffolds immerse in water for 1 h) weight of the scaffolds was measured.

$$\mathrm{porosity}\;\left[\%\right]=\frac{\left(\mathrm{scaffold}\;\mathrm{wet}\;\mathrm{weight}\;\left[g\right]-\;\mathrm{scaffold}\;\mathrm{dry}\;\mathrm{weight}\;\left[g\right]\right)}{\mathrm{scaffold}\;\mathrm{wet}\;\mathrm{weight}\;\left[g\right]}*100$$

$$\mathrm{swelling}\;\mathrm{ratio}\;\left[1\right]=\frac{\left(\mathrm{scaffold}\;\mathrm{wet}\;\mathrm{weight}\;\left[g\right]-\;\mathrm{scaffold}\;\mathrm{dry}\;\mathrm{weight}\;\left[g\right]\right)}{\mathrm{scaffold}\;\mathrm{dry}\;\mathrm{weight}\;\left[g\right]}$$

#### 2.2.4. Matrix Stiffness

As described before, Young’s modulus was used to calculate scaffold stiffness [36]. Briefly, scaffolds were compressed four times uniaxially by 10% of the original height. This cyclic compression was performed with a velocity of 5 mm/min using a ZwickiLine Z 2.5TN (Zwick GmbH & Co.KG, Ulm, Germany). A real-time Xforce HP 5N sensor measured the required load. Using the area and initial scaffold height the resulting load-deformation curve was translated into a stress-strain curve. The Young’s modulus in the region of linear elastic deformation is calculated as follows [37]:

$${\mathrm{Young}}^{’}s\;\mathrm{modulus}\;\left[\mathrm{MPa}\right]\;=\;\frac{\mathrm{applied}\;\mathrm{force}\;\left[N\right]*\mathrm{initial}\;\mathrm{scaffold}\;\mathrm{height}\;\left[\mathrm{mm}\right]}{\;\mathrm{area}\;\mathrm{of}\;\mathrm{the}\;\mathrm{scaffold}\;\left[{\mathrm{mm}}^{2}\right]*\mathrm{change}\;\mathrm{in}\;\mathrm{height}\;\left[\mathrm{mm}\right]}$$

### 2.3. Primary Human Ad-MSCs

#### 2.3.1. Ethics Statement

All investigations were conducted in accordance to the Declaration of Helsinki (1964). Cells used in this study were derived from adipose tissue of patients who were treated at our level 1 trauma center. In accordance with the ethical vote (539/2016BO2), tissue was only harvested after medical consultation and upon receipt of the patients’ written consent. The harvesting procedure had no influence on the orthopedic/trauma surgery, thus, patients had no additional risk for complications, which is in line with earlier reports [38]. Patients with viral/bacterial infections as well as patients not able to give their written consent were not included in the study.

#### 2.3.2. Donor Characteristics

The cells used in these experiments are from 5 donors: 2 males and 3 females. The average age is 61.2 years. One donor was a smoker and one donor was a diabetic. The average body mass index was 28.4 kg/m^2^ (19.7–33.4 kg/m^2^).

#### 2.3.3. Isolation and Expansion of Ad-MSCs

Fat tissues (obtained from hip or abdomen) were cut into mm-size pieces using a scalpel. The samples were washed several times with phosphate buffered saline (PBS). To release Ad-MSCs from the tissue samples were incubated in a 0.7% collagenase II solution (Biochrom, Berlin, Germany) for approximately 30 minutes at 37 °C. The addition of culture medium (DMEM 4.5 g/L glucose, 10% FCS, 100 U/mL penicillin, 100 µg/mL Streptomycin) stopped the collagenase II activity. After centrifugation at 600 g for 10 min the Ad-MSC pellet was re-suspended in culture medium for expansion (37 °C, 5% CO_2_, humidified atmosphere). Experiments were performed in passage 3 or 4 when Ad-MSCs were negative (determined by RT-PCR) for CD14 and positive for CD73, CD90, and CD105, as reported earlier [21,39].

#### 2.3.4. Sterilization of Scaffolds

For sterilization and to remove unreacted compounds (possible toxins) scaffolds were incubated for at least 12 h in 70% ethanol with agitation, followed by three washings steps (1, 6, and 12 h) with PBS. Sterilized scaffolds were stored at +4 °C until use. Prior to cell seeding, sterilized scaffolds were incubated in culture media for 24 h to calibrate scaffolds and ease cell attachment. Culture medium was removed from the scaffolds 1 h prior to cell seeding. Scaffolds were incubated at 37 °C for 1 h to reduce moisture and promote cell attachment.

#### 2.3.5. Cell Seeding

For expansion and plating, Ad-MSCs were detached from the culture flask with Trypsin/EDTA. Released cells were resuspended in culture medium at a concentration of approx. 2 × 10^6^ cells/mL. 30 µL of this cell suspension was placed centrally on top of each scaffold dropwise. After an initial incubation of 4 h in humidified atmosphere at 37 °C and 5% CO_2_, 136 μL of cell culture medium was carefully added. For complete adherence, the samples were incubated for 24 h in humidified atmosphere at 37 °C and 5% CO_2_.

#### 2.3.6. Osteogenic Differentiation of Ad-MSCs

For osteogenic differentiation, culture medium was replaced by osteogenic differentiation medium (DMEM 4.5 g/L glucose, 1% FCS, 200 μM L-ascorbic acid 2-phosphate, 5 mM β-glycerol phosphate, 25 mM HEPES, 1.5 mM CaCl_2_, 5 μM cholecalciferol, 100 U/mL penicillin, 100 µg/mL Streptomycin) for a period of 14 days [12]. Medium was changed every 2–3 days.

### 2.4. Functional Testing

#### 2.4.1. Mitochondrial Activity (Resazurin Conversion)

Scaffolds containing cells were washed once with PBS and then incubated with 0.0025% resazurin in plain culture medium. Scaffolds without cells were used as background control. After incubation for 2 h at 37 °C and 5% CO_2_ in humidified atmosphere the produced resorufin was measured by the fluorescence at 544 nm/590–10 nm using the Omega Plate Reader (BMG Labtech, Ortenberg, Germany) [40]. Therefore, 2 × 50 µL of each sample were transferred into cavities of fresh 96-well-plates.

#### 2.4.2. Life-Staining

Viable cells were visualized using the Calcein AM cell-permeable non-fluorescent dye which is converted into green fluorescent calcein by esterases in the cell cytoplasm. Nuclei were counterstained with Hoechst 33342. Cells were washed once with PBS before incubation with the staining solution (DMEM 4.5 g/L glucose, 2 µM Calcein AM, 0.001% Hoechst 33342) for 30 min at 37 °C and 5% CO_2_ in humidified atmosphere. Afterwards, cells were washed two times with PBS and fluorescence signals were immediately measured with a fluorescence microscope (Evos Fl).

#### 2.4.3. Glucose Consumption

Glucose consumption was determined 48 h after the media changes on day 0, 7, and 12. Culture supernatants were collected and frozen at −80 °C until measurement. Briefly, 15 µL sample was placed on parafilm to facilitate its resorption by the detection stripes (Contour next blood sugar meter sensor, Ascensia Diabetes Care Holdings, Basel, Switzerland). The level of consumption was measured by using a Contour XT blood sugar meter (Bayer Consumer Care, Basel, Switzerland). Glucose consumption was calculated by the difference of initial glucose concentration in the medium and the measured concentration in the sample.

#### 2.4.4. Total DNA Content

To isolate the total DNA of cells differentiated on the scaffolds, samples were washed once with PBS with the help of a cell strainer and centrifugation (600 g, ambient temperature, 10 min). Scaffolds without cells were used as background control. Scaffolds were incubated in 400 µL of 50 mM NaOH for 30 min at 98 °C. Following incubation, samples were vortexed and frozen at −80 °C for at least 1 h. After thawing 400 µL of a 100 mM Tris buffer (pH = 8.0) was added to each sample to neutralize pH. These samples were centrifuged at 14.000 g, at 4 °C for 10 min before supernatants were transferred into fresh reaction tubes. DNA concentration was determined photometrically using the LVIS plate and the Omega Plate Reader (BMG Labtech) [41].

#### 2.4.5. Alkaline Phosphatase (ALP) Activity

ALP activity was measured by incubating the scaffolds with cells (once washed with PBS) at 37 °C with 200 µL of substrate solution (1 mg/ml p-nitrophenyl phosphate, 50 mM glycine, 1 mM MgCl_2_, 100 mM TRIS, pH = 10.5). Scaffolds without cells were used as background control. After 40 min incubation at 37 °C and 5% CO_2_ in humidified atmosphere. 2 × 50 µL of each sample were transferred into cavities of fresh 96-well-plates. The enzymatically converted p-nitrophenol was photometrically measured at a wavelength of 405 nm with the Omega Plate Reader [40]. ALP activity was normalized to total DNA amounts.

#### 2.4.6. Dot Blot Analysis

To detect the intracellular osteogenic transcription factors Runt-related transcription factor 2 (Runx2) and osterix, cells were lysed with freshly prepared ice-cold RIPA buffer. To detect secreted osteoblast marker, e.g., osteocalcin, Osteoprotegerin (OPG) and receptor activator of nuclear factor kappa-Β ligand (RANKL), culture supernatants were analyzed. Next, 5 µL cell lysate/cell culture supernatant was placed on a dry nitrocellulose membrane. Membranes were blocked with 5% BSA in TBS-T for 1 h followed by overnight incubation at +4 °C with primary antibodies for RUNX2, osteocalcin, OPG, RANKL (sc-10758, sc-365797, sc-11383, sc-377079/Santa Cruz Biotechnology, Heidelberg, Germany), or osterix (MAB7547/R&D Systems, Minneapolis, MI, USA), diluted 1:1,000 in TBS-T. The next day, after washing the membranes were incubated with the corresponding peroxidase-labeled secondary antibodies (1:5000 in TBS-T/Santa Cruz Biotechnology) for 2 h. Signals were normalized to total DNA contents. For signal development, membranes were incubated for 1 min with ECL substrate solution. Chemiluminescent signals, detected by a CCD camera (INTAS, Göttingen, Germany), were quantified using the ImageJ software.

### 2.5. Statistical Analysis

Results are presented as violin plots or floating symbols (mean ± 95% confidence interval). Each experiment was performed with five donors (*N* = 5), with at least three replicates (*n* ≥ 3). Statistical analyses were performed using the GraphPad Prism Software (GraphPad, El Camino Real, USA). Groups were compared using the Kruskal–Wallis H-test followed by Dunn’s multiple comparison test. A *p*-value below 0.05 was considered statistically significant.

## 3. Results

### 3.1. pHEMA Concentration Affects Both Matrix Stiffness and Pore Size

**Figure 1:**
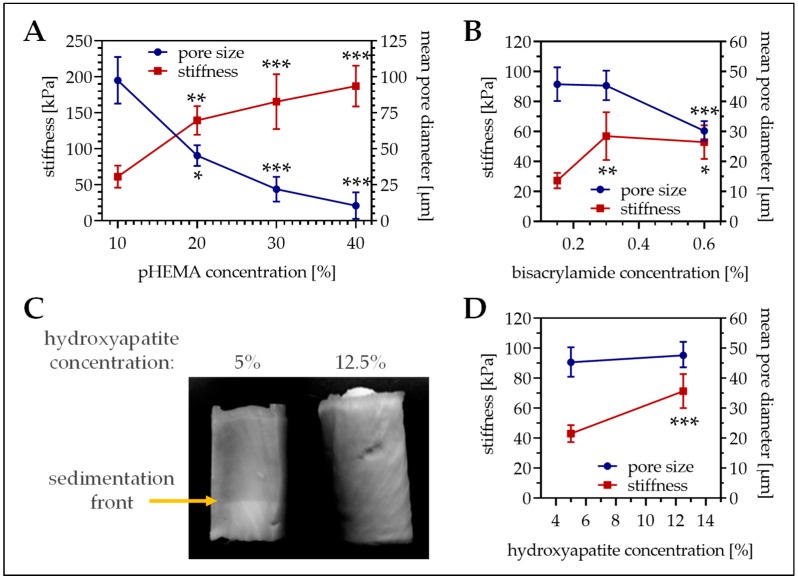
Pore size and stiffness of cryogels with different composition. (**A**) Variation of the pHEMA concentrations (10%, 20%, 30%, and 40% pHEMA with constant bisacrylamide ratio) in cryogels containing 5% hydroxyapatite. (**B**) Variation of the bisacrylamide concentration in 16% pHEMA cryogels containing 5% hydroxyapatite. (**C**,**D**) Variation of the hydroxyapatite concentration in 16% pHEMA (0.3% bisacrylamide) cryogels. (**C**) Representative photograph of the cryogel. (**A**,**B**,**D**) In the graphics pore diameter is represented with the blue line (right scale) and cryogel stiffness is represented with the red line (left scale). Single data points represent mean ± 95% C.I. * *p* < 0.05, ** *p* < 0.01, and *** *p* < 0.001 when compared to the lowest concentration presented.

First the optimal pHEMA concentration was determined. With increasing pHEMA concentrations the stiffness of the resulting cryogels (containing equal amounts of collagen and hydroxyapatite) increased, ranging from 10% pHEMA cryogels with a mean stiffness of 61.3 ± 15.4 kPa up to 40% pHEMA cryogels with a mean stiffness of 187.2 ± 28.3 kPa (Figure 1A, red line). Inversely, the mean pore diameter of the cryogels decreased with increasing pHEMA concentrations, ranging from 195.1 ± 32.5 µm for 10% pHEMA cryogels down to 21.0 ± 18.4 µm for 40% pHEMA cryogels (Figure 1A, blue line). To obtain both a large pore size and a high stiffness, further experiments were done with 16% pHEMA cryogels. To further increase stiffness of the cryogels (16% pHEMA), the bisacrylamide concentration was increased from 0.15% to 0.3% and 0.6%. Doubling the bisacrylamide concentration significantly increased the cryogel stiffness (Figure 1B, red line), without affecting the pore size (Figure 1B, blue line). Further increasing the bisacrylamide concentration could not additionally increase the cryogel stiffness but reduced the pore size. Thus, for all further experiments 16% pHEMA cryogels with 0.3% bisacrylamide as cross-linker were cast.

### 3.2. Calcium–Phosphate Crystallization on Cryogels Results in the Highest Stiffness without Affecting Pore Size

**Figure 2:**
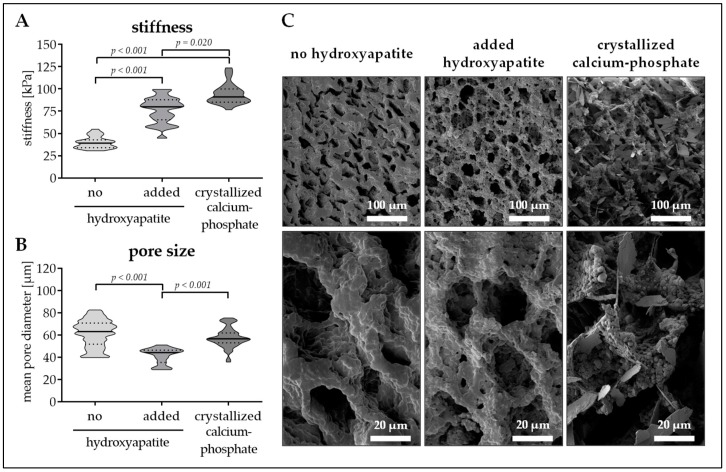
Pore size and stiffness of cryogels with or without hydroxyapatite. Cryogels containing 16% pHEMA (0.3% bisacrylamide, PRP) were supplemented with insoluble hydroxyapatite or with crystallized calcium–phosphate. (**A**) Stiffness of the resulting cryogels in kPa. (**B**) Pore diameter of the resulting cryogels in µm. Data are presented as violin plots (mean ± 95% C.I. is marked) of *N* = 5 (*n* = 3) individual experiments. (**C**) Scanning electron microscopy (SEM) images of the resulting cryogels.

To best mimic the bone inorganic matrix the cryogels should contain hydroxyapatite. We tested two different options. First insoluble hydroxyapatite was added to the cryogel. The hydroxyapatite was incorporated in the cryogel matrix, which increased matrix stiffness (39.9 ± 6.6 kPa vs. 76.6 ± 14.5 kPa, *p* < 0.001) but decreased pore size (61.5 ± 121 µm vs. 41.4 ± 7.0 µm, *p* < 0.001). Unfortunately, due to gravitational forces hydroxyapatite settled down during the polymerization process, such that a hydroxyapatite gradient arose (Figure 1C). To reduce gradient formation, the hydroxyapatite concentration had to be increased to 12.5%. This further increased cryogel stiffness (Figure 1D, red line), without affecting the pore size (Figure 1D, blue line). As a possible alternative, hydroxyapatite was crystallized from calcium and phosphate. For that method, a sodium dihydrogen phosphate buffer was added to the cryogel mixture before polymerization. After cutting, the still frozen cryogels are incubated in 1 M calcium chloride buffer for 24 h to facilitate hydroxyapatite crystallization. Interestingly, this method further increased cryogel stiffness (94.4 ± 11.5 kPa, *p* ≤ 0.02) without reducing the pore size (57.7 ± 8.8 µm/Figure 2A,B). SEM pictures revealed a roughening of the surface due to the addition of the hydroxyapatite, which was more pronounced with the calcium–phosphate crystallization method (Figure 2C). Therefore, cryogels for the following experiments were all made with calcium–phosphate crystallization method.

### 3.3. Altering the Protein Source in the Scaffold Affects Matrix Stiffness and Pore Size

In the next step, different protein additives have been included in the cryogels. As reference rat tail collagen (Coll) was chosen, as this represents the most common protein in bone. For better standardization, RGD-peptides (RGD), which are commonly used for material coating were chosen. Furthermore, PRP which represents a clinically used additive for synthetic bone filling material, as well as immune-cell conditioned medium (CM), which proved to be osteo-inductive in our previous studies, were used.

**Figure 3:**
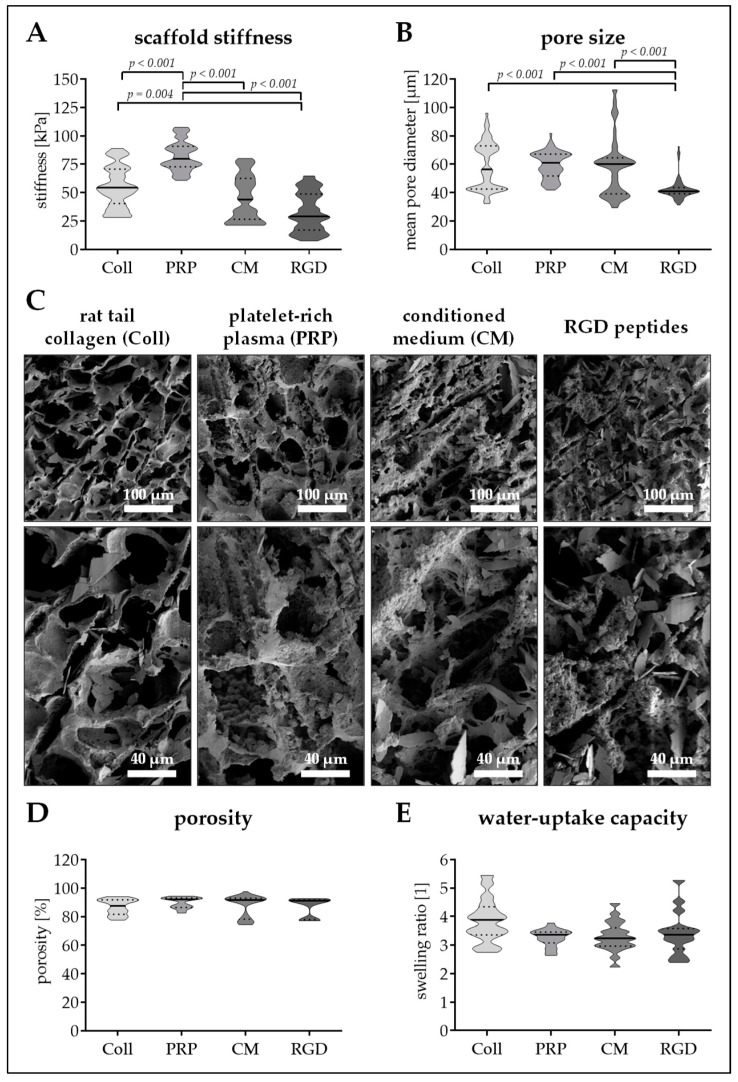
Pore size and stiffness of cryogels with different protein additives. Cryogels containing 16% pHEMA and crystallized calcium–phosphate were supplemented with different protein components: collagen (Coll), platelet-rich plasma (PRP), immune-cell conditioned medium (CM), and RGD peptides (RGD). (**A**) Stiffness of the resulting cryogels in kPa. (**B**) Pore diameter of the resulting cryogels in µm. (**C**) scanning electron microscopy (SEM) images of the resulting cryogels. (**D**) Scaffold porosity in %. (**E**) Water-uptake capacity of the scaffolds (fold of dry weight). Data are presented as violin plots (mean ± 95% C.I. is marked) of *N* = 5 (*n* = 4) individual experiments.

The different proteins strongly affected cryogel stiffness and pore size. Stiffness of the scaffolds increased in order of RGD, CM, Coll, and PRP. Cryogels containing PRP are characterized by a mean stiffness of 82.3 ±13.1 kPa, which is significantly (*p* < 0.001) higher than the other three scaffolds. Coll cryogels have the second highest stiffness with a young’s modulus of 55.3 ± 18.2 kPa, 42.8% less than that of PRP. CM and RGD cryogels have a stiffness of 45.6 ± 20.0 kPa and 32.8 ± 17.4 kPa, respectively (Figure 3A). With an average pore diameter of 59.4 ± 9.3 µm, PRP cryogels had the largest pores. These were comparable to the pores of Coll cryogels (58.9 ± 16.7 µm) and CM cryogels (58.1 ± 20.6 µm). Only RGD cryogels had significantly smaller pores (42.5 ± 7.5, *p* < 0.001/Figure 3B). SEM images support these findings, and show a rough cryogel surface with calcium phosphate (hydroxyapatite) crystals for all four scaffolds (Figure 3C).

Regarding porosity and water-uptake capacity, no significant differences between the four scaffolds could be detected. The mean porosity was between 87.0% (Coll cryogel) and 89.9% (PRP cryogel/Figure 3D), meaning, that the scaffold can take-up water 3.3- to 3.9-fold the weight of their dry mass (Figure 3E).

### 3.4. Seeding Efficiency and Cell Survival is Comparable between the Four Scaffolds

To provide optimal conditions for cell seeding, scaffolds were incubated in culture medium prior to the cell seeding process. Pre-incubation with culture medium for 24 h improved the cell attachment rate when compared to untreated cryogels. Further extending the pre-incubation period (2–7 days), however, failed to improve cell attachment as compared to the 24 h pre-incubation. Overall, a cell density of 1.5 to 2.5 × 10^6^ cells/ml proved to be most efficient for cell seeding as described in Materials and Methods.

**Figure 4:**
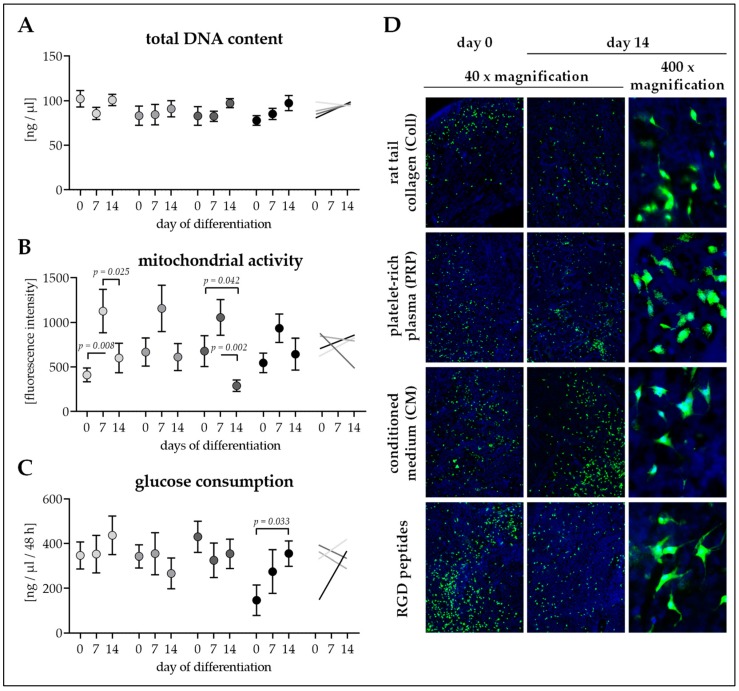
Cell attachment and survival on the four different cryogels. Cryogels containing 16% pHEMA, crystallized calcium–phosphate, and collagen (Coll), platelet-rich plasma (PRP), immune-cell conditioned medium (CM), or RGD peptides (RGD) were seeded with adipose-derived mesenchymal stem cells (Ad-MSCs). On days 0, 7, and 14 of osteogenic differentiation (**A**) total DNA content in ng/µl, (**B**) mitochondrial activity (resazurin conversion) in fluorescent intensities, and (**C**) glucose consumption (within 2 days) in ng/ml were determined. Data are presented as floating signs (mean ± 95% C.I.) of *N* = 5 (*n* ≤ 3) individual experiments. (**D**) Calcein–AM staining showing living cells in bright green on the cryogels. Hoechst was used as nuclear counterstain.

On days 0, 7, and 14 of osteogenic differentiation, cell attachment and viability were checked. At all time-points investigated total DNA content was comparable between the four scaffolds. Total DNA content remained constant over the differentiation period. Only total DNA content of Ad-MSCs on RGD cryogels showed an increasing trend (1.25-fold, *p* = 0.234) during the 14 days of osteogenic differentiation (Figure 4A).

Mitochondrial activity (resazurin conversion) was also comparable between the four scaffolds at all time-points investigated. For all four conditions, mitochondrial activity was highest at day 7 of osteogenic differentiation (Figure 4B), showing alterations in cellular metabolism throughout differentiation.

Glucose consumption was comparable between Ad-MSCs differentiated on the four scaffolds at all time-points investigated. Similar to the total DNA content, throughout the differentiation process glucose consumption remained constant for Ad-MSCs on Coll, PRP, and CM cryogels. Only Ad-MSCs on RGD cryogels showed increased glucose consumption (2.42-fold, *p* = 0.033) during the 14 days of osteogenic differentiation (Figure 4C).

Calcein-AM staining revealed an equal distribution of the cells on the cryogels from the time of seeding. Throughout the differentiation process, continuous viability of the cells remained constant. Cross sections of the scaffolds showed partial penetration of the cells into the scaffold (Figure 4D).

### 3.5. Ad-MSCs Differentiated on PRP Scaffolds Showed Best Osteogenic Characteristics

To measure the rate of osteogenic differentiation of the Ad-MSCs differentiating on the four scaffolds, expression of the two osteogenic transcription factors Runx2 (early) and osterix (late) was determined in cell lysates on days 0, 7, and 14 of differentiation. To assess resulting osteogenic function, ALP activity (early marker) and secretion of the osteogenic markers osteocalcin, OPG and RANKL were measured on days 0, 7, and 14 of differentiation.

**Figure 5:**
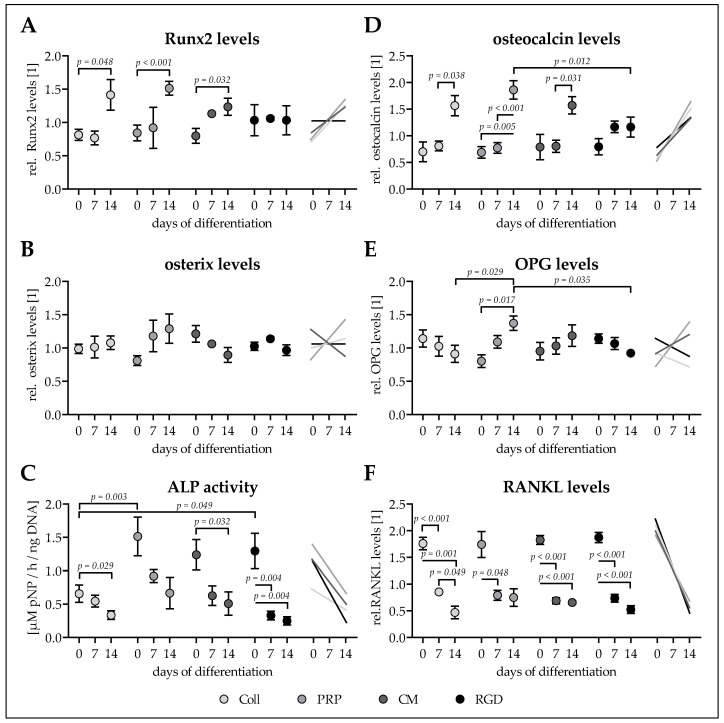
Osteogenic characteristics of Ad-MSCS differentiated for 14 days on the four different cryogels. Adipose-derived mesenchymal stem cells (Ad-MSCs) were osteogenically differentiated for 14 days on cryogels containing 16% pHEMA, crystallized calcium–phosphate, and collagen (Coll), platelet-rich plasma (PRP), immune-cell conditioned medium (CM), or RGD peptides (RGD). On days 0, 7, and 14 intracellular (**A**) Runt-related transcription factor 2 (Runx2) and (**B**) and osterix were determined by dot blot. (**C**) Alkaline phosphatase (ALP) activity on days 0, 7, and 14 of differentiation normalized to the total DNA contents. On days 0, 7, and 14 secreted levels of (**D**) osteocalcin, (**E**) osteoprotegerin (OPG), and (**F**) receptor activator of nuclear factor kappa-Β ligand (RANKL) were detected in the culture supernatants by dot blot. Data are presented as floating signs (mean ± 95% C.I.) of *N* ≥ 3 (*n* ≤ 3) individual experiments.

Basal Runx2 levels were comparable between all four conditions. Runx2 levels increased significantly with time only in Ad-MSCs differentiated on cryogels containing CM (1.55-fold, *p* = 0.032), Coll (1.74-fold, *p* = 0.048), and PRP (1.80-fold, *p* < 0.001), with the most pronounced increase in Ad-MSCs differentiated on PRP scaffolds (Figure 5A).

Similarly basal levels of osterix were comparable between all four conditions. Osterix levels showed a clear trend to increase only in Ad-MSCS differentiated on PRP cryogels (1.59-fold, *p* = 0.144). In Ad-MSCs differentiated on cryogels with Coll (1.09-fold) and RGD (0.94-fold) osterix levels remained equal. Interestingly, Ad-MSCs differentiated on cryogels with CM osterix levels showed a decreasing (0.70-fold, *p* = 0.412) trend (Figure 5B).

For all four conditions the early osteogenic marker ALP activity was overall highest on day 0 of differentiation. This was partly due to a very strong basal AP activity of two of the investigated donors. Interestingly, mean ALP activity was lower (0.43- to 0.53-fold) in Ad-MSCs seeded on Coll cryogels when compared to Ad-MSCs seeded on cryogels containing CM (*p* = 0.087), RGD (*p* = 0.049), or PRP (*p* = 0.003). With osteogenic differentiation the mean ALP activity decreased significantly in all four conditions (Figure 5C).

Basal osteocalcin secretion was comparable between all four conditions. Similar to Runx2 secreted osteocalcin levels increased significantly with time only in Ad-MSCs differentiated on cryogels containing Coll (2.24-fold, *p* = 0.038), PRP (2.70-fold, *p* = 0.005), and CM (1.99-fold, *p* = 0.031). The increase was again most pronounced in Ad-MSCs differentiated on PRP scaffolds, such that levels were significantly higher at day 14 (1.60-fold, *p* = 0.012) when compared to Ad-MSCs differentiated on RGD scaffolds (Figure 5D).

After cell seeding no significant differences in OPG secretion was detected across all four conditions. Interestingly, OPG secretion tended to decrease in Ad-MSCs differentiated on cryogels containing Coll (0.80-fold, *p* = 0.538) and RGD (0.81-fold, *p* = 0.077). On the contrary, OPG secretion tended to increase in Ad-MSCs differentiated on CM cryogels (1.24-fold, *p* = 0.540), an effect even significant in Ad-MSCs differentiated on PRP cryogels (1.71-fold, *p* = 0.017), resulting in OPG levels on day 14 being significantly higher than OPG levels in Ad-MSCs differentiated on Coll cryogels (1.51-fold, *p* = 0.029) or RGD cryogels (1.49-fold, *p* = 0.035/Figure 5E).

Similar to its antagonist OPG, secretion of RANKL was comparable between the four conditions right after cell seeding. In all four conditions RANKL secretion had already decreased significantly at day 7 of differentiation. The effect was most pronounced in Ad-MSCs differentiated on Coll cryogels (0.27-fold, *p* = 0.001), followed by Ad-MSCs differentiated on cryogels containing RGD (0.28-fold, *p* < 0.001), CM (0.36-fold, *p* < 0.001), and PRP (0.43-fold, *p* = 0.091) (Figure 5F).

## 4. Discussion

Bone tissue engineering aims at providing biologically active bone material for transplantation in large bone defects. Ideally, this engineered bone tissue contains the patients’ own stem cells on a carrier that mimics the extracellular matrix of bone. MSCs are the most promising cell type for this task, as they represent the most transplanted cell type [8]. Especially as regulations for transplantation are continuously tightened, mainly B-MSCs are used to generate these engineered bone tissues. With specific limitations in harvesting B-MSCs, Ad-MSCs represent a valuable alternative [9,10]. The osteogenic differentiation capacity of both B-MSCs and Ad-MSCs, however, depends on many factors. The most commonly described is the donors’ age [19]. Similar to an increased donor age, extended cell cultures reduce the osteogenic differentiation potential of MSCs. These age-dependent alterations in the differentiation capacity are strongly associated with epigenetic changes of the cells [17,18,20], e.g., a decreased expression of Nanog, Oct4α, and Lin28A suppressing the cells self-renewal capacity or increased expression of Sox2 suppressing the osteogenic differentiation [20]. Additionally, strong and continuous psychological stress may also hamper the osteogenic differentiation potential of MSCs [42]. Although some of these epigenetic alterations might be attenuated to a certain degree by small chemical inhibitors [20], their influence needs to be considered when using MSCs for bone tissue engineering. When using Ad-MSCs for bone tissue engineering, it is also necessary to consider the location of the fat tissue used for cell isolation. Just recently, we could show that Ad-MSCs derived from fat tissue of the hip, thigh or abdomen have a much stronger osteogenic differentiation capacity than Ad-MSCs derived from knee and limb [21]. In addition to these donor dependent factors, the actual carrier material and the culture conditions are equally essential for successful bone tissue engineering [22].

The utilized scaffolds should support the undifferentiated cells to either allow direct osteogenesis or to secrete paracrine factors. For bone tissue engineering a large number of different synthetic bioceramics as well as freeze-dried scaffolds exist [43,44,45,46]. Cultivation on three-dimensional scaffolds was shown to improve osteogenic properties of Ad-MSCs [47,48], amongst others through control of cell agglomeration [49], a factor critically dependent on the scaffolds’ pore size. These studies have shown that scaffolds containing hydroxyapatite, the major mineral component of bone, often favor cell attachment, proliferation, and osteogenic differentiation [43,50]. In this study we generated cryogels based on the polymer pHEMA, which has been already used for a plethora of biomedical applications due to its good biocompatibility and tunable mechanical properties [31,51,52,53,54]. We induce polymerization and cross-linking with bisacrylamide, glutaraldehyde, APS, and TEMED, which are toxic to cells in soluble form. However, in the interconnected solid form the polymerized material is nontoxic [34,55]. To assure compatibility with the cells, possible remaining unreacted ingredients were removed by the washing and disinfecting steps before use in vitro. We optimized the pHEMA concentration and bisacrylamide crosslinking so that both the scaffold stiffness and the pore size remained as large as possible. Incorporation of the insoluble hydroxyapatite into the cryogel significantly increased the cryogel stiffness but decreased the pore size. Furthermore, gravitational forces resulted in sedimentation of the hydroxyapatite, such that a hydroxyapatite gradient developed within the scaffolds. Thus, we tested calcium–phosphate crystallization on the formed cryogels. For that reason, a sodium dihydrogen phosphate buffer was added to the cryogel mixture prior to freezing. The frozen cryogels were then incubated in a CaCl_2_ buffer to allow calcium–phosphate crystal formation during the thawing process. This method not only further increased the cryogel stiffness, but also increased the pore size of the scaffolds as compared to the scaffolds with added insoluble hydroxyapatite. This effect might be explained by an increased freezing point due to the sodium ions. Unfortunately, changing the protein source for our cryogels had significant effects on the pore size and stiffness of the resulting scaffolds. Thus, the observed effects cannot be exclusively addressed to the used protein source.

With a mean stiffness of ~55 kPa our Coll cryogel is more rigid than most common scaffolds based on collagen, which are mostly flexible [25]. Sun et al. showed that a carrier stiffness > 60 kPa induces expression of osteogenic transcription factors and marker genes [56,57], which holds for our PRP cryogel. However, lowering the carrier stiffness increased expression of stem cells markers, among other also Sox2, which proved to effectively inhibit osteogenic differentiation [58,59,60,61]. Alexander et al. proposed high expression of tissue non-specific ALP to be favorable for osteogenic differentiation of stem cells [62]. But high ALP activity in stem cells is not only attributed to the tissue non-specific ALP, but also to other members of the ALP family [63]. In addition, there is continuous discussion, that low carrier stiffness might even favor MSC differentiation toward adipocytes [26]. Furthermore, the calcium–phosphate crystallization method additionally increased the surface roughness of the scaffold. The work of Faia-Torres, et al. clearly shows the influence of the surface roughness on the osteogenic differentiation potential of MSC [64,65]. In their studies increasing the surface roughness was sufficient to induce spontaneous osteogenesis. Mathieu et al. postulate that osteogenic differentiation is triggered because the cells strongly develop focal adhesions [66]. This might explain why rougher surfaces favor osteogenic differentiation.

With a mean pore diameter of almost 60 µm our cryogels are at the lower end of the recommended pore size for osteogenic differentiation, which is in the range of 20 µm to 300 µm [43]. It is postulated that smaller pores may hamper vascularization when transplanted [67]. Thus, further studies claim a minimum pore size of 100 µm for successful ingrowth of bone cells into scaffolds [68,69]. Although the mean pore size of our scaffolds is below that limit, we could show agglomeration and partial ingrowth of the Ad-MSCs. Which is supported by the study of Kim et al. showing that cryogel based filling material with a pore size as small as 10 µm improved healing of large bone defects in rabbits [32].

Interestingly, altering the protein additives within our scaffolds strongly affected the scaffold stiffness and pore size. In this study, four different protein additives have been compared, which proved to induce osteogenic capacity in earlier studies. Collagen, being the most abundant organic matrix in bone, represents the most commonly used protein carrier for bone scaffolds. Therefore, rat tail collagen was used for a control scaffold. With a mean pore size (58.9 µm) and stiffness (55.3 kPa), the resulting cryogels are within the range suggested to be osteo-inductive [43,56]. Ad-MSCs differentiated on these scaffolds showed increasing expression of most osteogenic transcription factors and markers investigated. ALP activity varied strongly between the investigated donors. Overall, mean ALP activity decreased with differentiation time, which is common for all four scaffolds.

The four scaffolds have in common that they contain reasonable amounts of organic and inorganic (calcium–phosphate) matrix. Cells cultured on these scaffolds might not further induce ALP expression as observed in conventional 2D cultures, where organic matrix has to be first produced and then mineralized with the help of the ALP. Therefore, it is feasible that the drop in ALP activity observed later in osteogenic differentiation, occurs faster in these 3D cultures when compared to osteogenic differentiation in conventional 2D cultures [21]. Furthermore, ALP activity has been described to be strongly affected by cell density and the time of cultivation [70]. Therefore, formation of cell agglomerates on the scaffolds might affect ALP activity as well.

As collagen is often obtained from animals, and thus requires special approval for clinical use, we replaced collagen with synthetic RGD-peptides, which represent the most common cell attachment motive in the extracellular matrix. The synthetic RGD-peptides have the great advantage of reproducibility. Furthermore, in vitro studies showed that RGD coatings may improve cell attachment and support osteogenic differentiation [71,72,73]. As expected, the resulting cryogels were most reproducible. Unfortunately, replacing the collagen with RGD significantly lowered both the cryogel stiffness (−40%) and pore size (−27%). Although this did not affect cell attachment it seemed to slightly induce cell proliferation despite the osteogenic stimuli. Basal ALP activity was higher for Ad-MSCs on RGD cryogels when compared to Ad-MSCs on Coll cryogels. As ALP activity is also a characteristics of stem cells [63], it is feasible that with the reduced stiffness of the scaffold, which preserved stem cell characteristics of MSCs in the study of Sun et al. [56], basal ALP activity is elevated. The reduced pore size might explain why Ad-MSCs differentiated on these scaffolds showed lowest expression of osteogenic transcription factors and markers with time [68,69].

In previous studies, co-cultivation of Ad-MSCs with endothelial cells, vascular cells or osteoblastic cells favored their osteogenic differentiation [74,75,76]. It is thought that the additional cells provide chemokines and growth factors required for osteogenesis. We could show strong osteogenic effects of CM not only on migration and proliferation [7] of osteogenic cells, but also on their function. Therefore, we used CM as an additional protein source. Replacing Coll with CM did not significantly alter stiffness or pore size of the resulting cryogels. Cell attachment and survival was comparable between the two scaffolds. Even expression of Runx2, osteocalcin and RANKL was comparable between both scaffolds. Only OPG expression showed an inverse trend. While Ad-MSCs differentiated on Coll cryogels expressed less OPG over time, Ad-MSCs differentiated on CM cryogels expressed more OPG. Under this condition, OPG expression might be due to the cytokines secreted by the immune cells [77]. Again basal ALP activity was higher Ad-MSCs on CM cryogels when compared to Ad-MSCs on Coll cryogels. Therefore, the resulting drop with differentiation was more pronounced under this condition.

In conventional 2D cultures, supplementation of medium with platelet lysate or PRP was reported to improve osteogenic differentiation of Ad-MSCs [14,78,79]. Therefore, PRP was used as an alternative protein carrier. Interestingly, replacing collagen with PRP not only increased the cryogel stiffness (+39%), but also resulted in a more homogeneous pore size. The resulting PRP cryogels thus have a stiffness very close to the stiffness of collagenous bone [57,80]. Cell attachment and survival was comparable between Coll and PRP scaffolds. When compared to Ad-MSCs differentiated on Coll cryogels, Ad-MSCs differentiated on PRP cryogels expressed even higher levels of the investigated osteogenic markers. Interestingly, OPG expression was even higher than that in Ad-MSCs differentiated on CM cryogels, supporting the idea of higher cytokine levels in PRP cryogels than in CM cryogels which favor OPG expression [77]. ALP activity in these cells was comparable to Ad-MSCs differentiated on RGD and CM cryogels.

## 5. Conclusions

In summary, all four cryogels displayed good cell attachment and survival. The very soft RGD cryogels, were highly homogeneous and seemed to favor cell proliferation. Whether the reduction in stiffness preserves stem cell characteristics needs to be further investigated. CM scaffolds showed comparable results to Coll scaffolds, however, due to the higher heterogeneity the classical Coll cryogels might be favorable. Noteworthy, our PRP cryogels showed better physical characteristics (stiffness and pore size) than the Coll cryogels, resulting in better functional measures, e.g., ALP activity and expression of osteogenic markers. Therefore, this cryogel constitutes a suitable carrier for bone tissue engineering, especially as this scaffold can be generated without animal products but with the patients’ own PRP.

## Figures and Tables

**Table 1:** Composition of the different cryogel-scaffolds. Final concentrations are given (ddH_2_O was used to adjust the volume).

	w/o HA	w/ HA	Coll	PRP	RGD	CM
**pHEMA**	16.0%	16.0%	16.0%	16.0%	16.0%	16.0%
**bisacrylamide**	0.3%	0.3%	0.3%	0.3%	0.3%	0.3%
platelet rich plasma (PRP) *	0.25 g/L	0.25 g/L	-	0.25 g/L	-	-
**rat tail collagen (Coll) [33]**	-	-	0.25 g/L	-	-	-
**THP-1 cell conditioned medium (CM) ^#^ [7]**	-	-	-	-	-	1.0%
**RDG Peptide (RGD) °**	-	-	-	-	0.5 µM	-
**hydroxyapatite (HA)**	-	12.5%	-	-	-	-
**sodium dihydrogen phosphate**	-	-	0.3 M	0.3 M	0.3 M	0.3 M
**glutaraldehyde**	0.1%	0.1%	0.1%	0.1%	0.1%	0.1%
**ammonium persulfate (** **APS)**	0.2%	0.2%	0.2%	0.2%	0.2%	0.2%
**tetramethylethylenediamine (** **TEMED)**	0.2%	0.2%	0.2%	0.2%	0.2%	0.2%

* prepared by centrifugation (1000 g, 10 min) from EDTA blood of volunteers (pool of ≥ 5 donors); ^#^ CM medium was generated by culturing 4 × 10^5^ THP-1 cells per ml RPMI 1640 media (5% FCS, 100 U/mL penicillin, 100 µg/mL Streptomycin) for 2 days (37 °C, 5% CO_2_, humidified atmosphere). Cells were removed by centrifugation at 600 g for 10 min. CM was stored at −80 °C until use [7]; ° RGD peptides (H-Arg-Gly-Asp-OH) were obtained from Bachem (Bubendorf, Switzerland); w/o—without; w/—with.
